# Projected Impact of Mexico’s Sugar-Sweetened Beverage Tax Policy on Diabetes and Cardiovascular Disease: A Modeling Study

**DOI:** 10.1371/journal.pmed.1002158

**Published:** 2016-11-01

**Authors:** Luz Maria Sánchez-Romero, Joanne Penko, Pamela G. Coxson, Alicia Fernández, Antoinette Mason, Andrew E. Moran, Leticia Ávila-Burgos, Michelle Odden, Simón Barquera, Kirsten Bibbins-Domingo

**Affiliations:** 1 Center for Nutrition and Health Research, National Institute of Public Health, Cuernavaca, Morelos, Mexico; 2 Department of Epidemiology and Public Health, University College London, London, United Kingdom; 3 Department of Medicine, University of California, San Francisco, San Francisco, California, United States of America; 4 UCSF Center for Vulnerable Populations at San Francisco General Hospital, San Francisco, California, United States of America; 5 Division of General Internal Medicine, Columbia University Medical Center, New York, New York, United States of America; 6 Center for Health Systems Research, National Institute of Public Health, Cuernavaca, Morelos, Mexico; 7 Department of Epidemiology, Oregon State University, Corvalis, Oregon, United States of America; 8 Department of Epidemiology and Biostatistics, University of California, San Francisco, San Francisco, California, United States of America; University of Otago, Wellington, NEW ZEALAND

## Abstract

**Background:**

Rates of diabetes in Mexico are among the highest worldwide. In 2014, Mexico instituted a nationwide tax on sugar-sweetened beverages (SSBs) in order to reduce the high level of SSB consumption, a preventable cause of diabetes and cardiovascular disease (CVD). We used an established computer simulation model of CVD and country-specific data on demographics, epidemiology, SSB consumption, and short-term changes in consumption following the SSB tax in order to project potential long-range health and economic impacts of SSB taxation in Mexico.

**Methods and Findings:**

We used the Cardiovascular Disease Policy Model–Mexico, a state transition model of Mexican adults aged 35–94 y, to project the potential future effects of reduced SSB intake on diabetes incidence, CVD events, direct diabetes healthcare costs, and mortality over 10 y. Model inputs included short-term changes in SSB consumption in response to taxation (price elasticity) and data from government and market research surveys and public healthcare institutions. Two main scenarios were modeled: a 10% reduction in SSB consumption (corresponding to the reduction observed after tax implementation) and a 20% reduction in SSB consumption (possible with increases in taxation levels and/or additional measures to curb consumption). Given uncertainty about the degree to which Mexicans will replace calories from SSBs with calories from other sources, we evaluated a range of values for calorie compensation.

We projected that a 10% reduction in SSB consumption with 39% calorie compensation among Mexican adults would result in about 189,300 (95% uncertainty interval [UI] 155,400–218,100) fewer incident type 2 diabetes cases, 20,400 fewer incident strokes and myocardial infarctions, and 18,900 fewer deaths occurring from 2013 to 2022. This scenario predicts that the SSB tax could save Mexico 983 million international dollars (95% UI $769 million–$1,173 million). The largest relative and absolute reductions in diabetes and CVD events occurred in the youngest age group modeled (35–44 y).

This study’s strengths include the use of an established mathematical model of CVD and use of contemporary Mexican vital statistics, data from health surveys, healthcare costs, and SSB price elasticity estimates as well as probabilistic and deterministic sensitivity analyses to account for uncertainty. The limitations of the study include reliance on US-based studies for certain inputs where Mexico-specific data were lacking (specifically the associations between risk factors and CVD outcomes [from the Framingham Heart Study] and SSB calorie compensation assumptions), limited data on healthcare costs other than those related to diabetes, and lack of information on long-term SSB price elasticity that is specific to geographic and economic subgroups.

**Conclusions:**

Mexico’s high diabetes prevalence represents a public health crisis. While the long-term impact of Mexico’s SSB tax is not yet known, these projections, based on observed consumption reductions, suggest that Mexico’s SSB tax may substantially decrease morbidity and mortality from diabetes and CVD while reducing healthcare costs.

## Abstract 2

Using consumption trends following the implementation of Mexico's tax on sugar sweetened beverages, Kirsten Bibbins-Domingo and colleagues estimate the tax's impact on diabetes cases, cardiovascular events, mortality and healthcare costs over the next ten years.

## Author Summary

**Why Was This Study Done?:**

- The prevalence of obesity and diabetes in Mexico has risen dramatically in recent years, and the rate of diabetes in Mexico currently ranks among the highest in the world.
- Mexicans consume a large volume of sugar-sweetened beverages (SSBs), a preventable risk factor for obesity, diabetes, and cardiovascular diseases.
- In order to address the obesity and diabetes epidemic, the Mexican government implemented a 10% excise tax on SSBs in 2014.
- Although consumer data suggest that the tax has resulted in a decrease in SSB purchases, little is known about the longer-term impact the SSB tax will have on disease burden and healthcare costs in Mexico.

**What Did the Researchers Do and Find?:**

- We conducted a computer simulation study in order to understand the potential impact of the tax on diabetes, cardiovascular diseases, mortality, and healthcare costs associated with diabetes among Mexican adults 35–94 years of age over a period of 10 years (2013–2022).
- To do this, we developed a Mexico version of an established model of cardiovascular disease in the US (the Cardiovascular Disease Policy Model) using national-level data from Mexico wherever possible.
- We found that the 10% tax on SSBs will likely prevent approximately 189,300 new cases of type 2 diabetes, 20,400 incident strokes and heart attacks, and 18,900 deaths over 10 years among adults 35–94 years of age, and is expected to result in 983 million international dollars in savings in healthcare costs because of the prevention of diabetes cases.
- The largest reductions in health burden and healthcare spending are projected to occur among the youngest adults included in the simulation (those 35–44 years of age).

**What Do These Findings Mean?:**

- The national tax on SSBs in Mexico is projected to have a substantial impact on the burden of diabetes, cardiovascular diseases, and mortality over 10 years.
- With the largest effects of the tax observed among the youngest age group modeled, we expect the impact of the intervention to become more dramatic as the population ages.
- The SSB tax may be an important component in a multifaceted strategy by the Mexican government to curb the obesity and diabetes epidemic in Mexico.

## Introduction

Sugar-sweetened beverage (SSB) consumption has risen rapidly in Mexico in recent years [1,2], with one-fifth of total daily caloric intake among adults currently estimated to come from SSBs (including sugar-sweetened soda, coffee, tea, and *agua fresca* [flavored water]). Observational and intervention studies have demonstrated that higher SSB consumption is linked to greater risk of cardiometabolic outcomes [3–5]. Of particular concern in Mexico is the link between high SSB consumption and obesity and diabetes given the dramatic increase in these conditions over a short period of time [6]. The prevalence of obesity and diabetes in Mexico now rank among the highest in the world; approximately 70% of Mexican adults are considered overweight or obese, and 14% have diabetes [7]. Diabetes is now the leading cause of death and, in 2011, cost the Mexican government an estimated US$7.7 billion in direct and indirect costs [8,9]. As a result of recent trends, Mexico is projected to bear an ever increasing burden of health-related costs from diabetic complications including cardiovascular disease (CVD).

In response to the population’s high consumption of SSBs and the diabetes epidemic, the Mexican government instituted several initiatives in 2014 guided by recommendations from its Ministry of Health [10]. Included among these initiatives was a 10% excise tax on SSBs (one Mexican peso per liter) levied on manufacturers effective January 1, 2014. Market research surveys have demonstrated that SSB purchases by Mexican households declined by an average of 6% in 2014, reaching a 12% decrease by December 2014 [11]. The expectation is that taxation will result in sustained population-wide reductions in SSB intake and downstream prevention of diabetes, CVD, and other negative health outcomes. However, epidemiologic and economic evidence demonstrating the policy’s impact will take years to emerge and may be confounded by changes in medical practice and other secular trends. The purpose of this analysis is to build on current evidence in order to project the longer-term impact of SSB taxation on diabetes, coronary heart disease (CHD), stroke, mortality, and associated healthcare costs in Mexico.

## Methods

### The Cardiovascular Disease Policy Model developed for Mexico

The Cardiovascular Disease Policy Model (CVDPM) is a computer-simulation state transition (Markov) model of CHD and stroke incidence, prevalence, mortality, and costs and has been used to forecast trends in CVD in the US and other countries for over 25 y [12–14]. The CVDPM models the population of adults aged 35–94 y (and excludes children and young adults under 35 y of age), with new 35-y-olds entering the simulated population each annual cycle. The CVDPM uses estimates generated from Framingham Heart Study Original Cohort and Offspring Cohort data [15–18] to predict incident type 2 diabetes, CHD, stroke, and non-CVD mortality among persons without CVD, based on age, sex, systolic blood pressure (SBP), body mass index (BMI), high-density lipoprotein cholesterol level, low-density lipoprotein cholesterol level, smoking, and type 2 diabetes mellitus. The CVDPM also predicts subsequent life years, CVD events, coronary revascularization procedures, CVD mortality, and non-CVD mortality among persons with CVD. The model is written in Fortran 95 and compiled using the Lahey Fortran 95 compiler V7.2 (Lahey Computer Systems).

The present analysis uses a newly created version of the CVDPM, the CVDPM–Mexico, adapted for analyses of epidemiology and policies in the Mexican population. The CVDPM–Mexico is based on Mexico-specific demographic and epidemiologic data and is calibrated to cardiac events and deaths among Mexicans in 2010. The CVDPM–Mexico is summarized below, and a detailed description is presented in S1 Appendix, which includes a description of the model’s structure (Figure A in S1 Appendix), data sources for Mexico-specific inputs (Table A in S1 Appendix), model calibration (Figure B in S1 Appendix), and intervention simulation assumptions (Tables B and C and Figure C in S1 Appendix).

### Mexico-Specific Epidemiologic Inputs

Estimates of the 2010 adult population of Mexico were obtained from the National Institute of Statistics and Geography (Instituto Nacional de Estadística y Geografía), and projections of the population through 2050 from the National Population Council (Consejo Nacional de Población) [19,20].

Age- and sex-specific means and distributions for five of six modeled risk factors (SBP, low-density lipoprotein cholesterol, high-density lipoprotein cholesterol, BMI, and diabetes) were estimated from the 2006 Mexican National Health and Nutrition Survey (Encuesta Nacional de Salud y Nutrición [ENSANUT]) [21], a nationally representative cross-sectional survey that used multi-stage cluster sampling of the Mexican population. Data on smoking prevalence was obtained from the 2009 Adult Smoking Survey (Encuesta Global de Tabaquismo en Adultos) [22], a nationally representative survey of Mexicans aged 15 y and older. Estimates of CHD and stroke prevalence were generated from ENSANUT 2006. Data on total and cause-specific mortality in 2010 are from the National Health Information System (Sistema Nacional de Información en Salud [SINAIS]) [23], and deaths were categorized according to the International Classification of Diseases (ICD-10) codes [24].

National in-hospital CHD and stroke events, deaths, and case fatality rates in 2010 were estimated based on data from three public health institutions that provide health services to 73% of Mexico’s population [7]: the Mexican Social Security Institute (Instituto Mexicano del Seguro Social [IMSS]) [25], the State Employee’s Social Security and Services Institute (Instituto de Seguridad y Servicios Sociales de los Trabajadores del Estado [ISSSTE]) [26], and the Ministry of Health (data acquired from SINAIS) [27,28]. Deaths occurring in other public health institutions were obtained from SINAIS 2010 mortality data [29]. The CVDPM–Mexico was calibrated to within ≤1% of national estimates of CHD and stroke events and CHD deaths in 2010 (Figure B in S1 Appendix).

### Mexico-Specific Healthcare Costs

We computed age-decile-specific mean annual direct per-patient healthcare costs incurred by persons with type 2 diabetes in 2012 using methodology described previously and detailed in S1 Appendix [30,31]. We first used national expenditure data from the System of Health Accounts (Sistema de Cuentas de Salud en Mexico) [32] to determine the total 2011 expenditures separately for generalist outpatient visits, specialist outpatient visits, and hospitalizations for each of the three primary healthcare institutions (IMSS, ISSSTE, and the Ministry of Health). We then determined the average cost of each visit type for each institution by dividing the expenditure total by the count of visits and hospitalization days obtained from utilization databases [25,26,28]. Utilization data were further used to determine the average number of services used in 2011 by those with diabetes (defined as records with ICD-10 codes E11–E14 in the first or second code position) for each institution, service type, and age stratum; these values were then multiplied by the institution-specific average cost for each service and summed over service types for institution-level annual costs per diabetes patient by age. For our cost inputs, we then generated an overall mean annual cost per diabetes patient for each age group (model cost inputs) by taking a weighted average of institution-specific costs, using as weights the proportion of diabetes patients receiving healthcare at each institution as measured by ENSANUT 2012 [7]. Expenditures were converted into 2011 US dollars using a purchasing power parity rate of 7.67 Mexican pesos = 1.00 international dollar, and inflated to 2012 currency using a 3.57% inflation rate [33]. The total direct healthcare expenditure calculated for Mexicans aged 35 to 94 y and living with diabetes in 2011 was approximately 7 billion international dollars (54 billion Mexican pesos) (Table B in S1 Appendix).

### Base Case Sugar-Sweetened Beverage Consumption in Mexico

Data from ENSANUT 2012 were used to estimate daily caloric intake from SSBs [7]. A one-time 24-h dietary recall administered to 13% of the ENSANUT 2012 sample assessed the type and quantity of foods and beverages consumed over the past day during meals and between meals, occurring both inside and outside the home, including measurement on weekdays and weekends [7,34]. The following were included in our definition of SSBs: industrialized flavored waters, sodas, *aguas frescas*, *horchatas* (rice waters), chocolate water, fruit juices, vegetable juices, and sports and energy drinks. Because interventions simulated in the CVDPM operate as changes in age- and gender-stratified mean values, we computed the mean and standard error (SE) of total volume (in milliliters) and number of servings (355 ml [12 fl. oz.] = 1 serving) of SSBs consumed per day for each sex and age stratum, which were in turn used to generate model inputs. The number of servings per day was also converted into average total calories from SSBs consumed per day (1 serving = 150 calories).

### Effect of Sugar-Sweetened Beverages on Cardiovascular Disease Risk Factors

Based on review of epidemiologic studies, we modeled direct and independent effects of SSB consumption on three factors related to CVD: diabetes incidence, mean BMI, and mean SBP (Figure C in S1 Appendix) [5,35]. In addition, changes in diabetes incidence and mean SBP associated with SSB intake were assumed to be partially mediated through BMI [5,36]. Each additional serving of SSB per day (defined as 355 ml/d) was assumed to be associated with a 1.19-fold (95% CI 1.09, 1.31) increased risk of diabetes independent of changes in BMI [5]. Inputs for the increased risk of diabetes associated with increased BMI were estimated using Framingham Heart Study data [15–18]. For blood pressure, we assumed that each additional SSB serving per day is associated with an SBP increase of 0.78 mm Hg (95% CI 0.09, 1.47) in men and 0.61 mm Hg (95% CI −0.27, 1.48) in women independent of BMI and that each one-unit increase in BMI is associated with a 1.43–mm Hg and 1.24–mm Hg increase in SBP in men and women, respectively [35]. Changes in caloric consumption were translated into changes in weight (using the conversion of 3,500 kcal/lb.), and the average height reported in ENSANUT 2006 was used to calculate corresponding changes in BMI (weight [kg]/height [m]^2^). Because the relationship between caloric consumption and weight loss may be variable and because it is not precisely known the degree to which calories reduced through decreased SSB consumption will be replaced by calories from other sources [37,38], we include model scenarios that assume the broadest possible range in weight change (from no weight change associated with SSB consumption change to all calories reduced through decreased SSB consumption translated into weight change as described above). For a detailed description of our approach to modeling the relationship between changes in SSB consumption and CVD risk factors and outcomes, see our previous work on SSBs in the US [39,40].

### Model Simulations

The CVDPM–Mexico was used to predict the impact of reductions in SSB intake on type 2 diabetes incidence, CVD incidence, CVD mortality, all-cause mortality, and healthcare costs over 10 y (2013–2022). To quantify anticipated changes in CVD risk factors expected from the SSB tax, two factors were taken into consideration: (1) the change in SSB consumption predicted to result from the 10% excise tax (price elasticity) and (2) the degree to which individuals would replace calories consumed through SSBs with calories from other sources (calorie compensation). SSB purchases by Mexican households declined by an increasing rate over 2014, reaching a decrease of 12% by December 2014 [11]. This observed effect corresponds with studies of Mexico’s consumer soda price elasticity (−0.72 to −1.30), suggesting that the 10% tax on SSBs in Mexico would result in a 10%–13% decline in SSB consumption [41,42]. Our main analysis modeled a 10% decrease in SSB consumption starting in year 1 and sustained over the 10-y simulation. Colchero et al. observed an average increase in purchases of non-taxed beverages including diet soda, water, and unsweetened dairy and fruit juice in response to the tax, but whether this shift in consumption resulted in a reduction in calories consumed is unclear [11]. We therefore estimated that 39% of the calories reduced by lowering SSB intake would be replaced by calories from other sources, yielding a net 61% reduction in total calories from SSBs, based on prior work in the US context [38]. The sensitivity of results to this assumption were tested in separate analyses that assumed 0% caloric compensation (all calories reduced through decreased SSB consumption are translated into weight changes) and 100% caloric compensation (only the independent effects of decreased SSB intake on diabetes and SBP are modeled, with no changes in BMI). In addition to assuming a 10% decline in SSB consumption for primary analyses, additional simulations were performed assuming a 20% reduction in SSB intake with 0%, 39%, and 100% calorie compensation in order to evaluate benefits that might be achieved from additional interventions such as higher levels of SSB taxation or other initiatives that have been implemented by the Mexican government, including nutritional guidelines for school lunches, front-of-package food labeling, and restrictions of food and beverage advertising targeted to children [10,43].

### Probabilistic and Deterministic Sensitivity Analyses

We used Monte Carlo simulations to generate 95% uncertainty intervals (UIs) around our primary outcome measures for each intervention scenario. The 95% confidence intervals for estimates of the effect of changes in SSB consumption on SBP and on diabetes risk as well as the beta inputs for model risk functions defining the relationship between risk factors and incident diabetes, incident CHD, incident stroke, and non-CVD death are included in Table C of S1 Appendix. There were 1,000 random draws from a standard normal distribution, scaled to the mean and confidence interval, for each varied parameter. The Monte Carlo program, written in Python, generated a new set of input parameters drawn from the distributions for each iteration, ran the given iteration base case and reduced SSB consumption simulations with the new parameters, and stored the outcomes for each iteration. The 95% UIs for each outcome were then calculated using Microsoft Excel 2010.

We used deterministic approaches to evaluate the sensitivity of our results to assumptions about the degree of calorie compensation and weight loss associated with reduced SSB intake (described above). To assess the health and economic impacts that could result from higher levels of taxation and/or additional public health interventions to curb SSB consumption in Mexico, we conducted a scenario analysis that assumed a 40% reduction in current levels of SSB consumption. There is a limited body of evidence suggesting that the risk of CHD, stroke, and non-CVD death may be higher among individuals with diabetes in Mexico compared to individuals with diabetes in the US [44,45]. We therefore conducted a sensitivity analysis using betas approximately 2-fold higher than those derived from the Framingham Heart Study and evaluated the effect on CVD and mortality outcomes.

## Results

Mexican men consumed an average of 1.24 (SE = 0.07) servings of SSBs per day in 2012, and women consumed an average of 0.86 (SE = 0.04) servings per day (Table 1). Men and women consumed a median 0.97 (interquartile range 0–1.68) and 0.64 (interquartile range 0–1.41) servings per day, respectively (S1 Table). Overall, younger adults had higher SSB consumption than older adults. Assuming no change in SSB consumption from that observed in 2012 and assuming current obesity rates, model simulations project 3.9 million new cases of diabetes and 1.2 million CVD deaths occurring among adults 35 to 94 y old between 2013 and 2022.

**Table 1:** Mean total volume and number of servings of sugar sweetened beverages consumed per person per day among Mexican adults from the 2012 Mexican National Health and Nutrition Survey (ENSANUT).

Age Group	Men				Women			
	*n* ^a^	*N* ^b^	Mean Daily SSB Consumption (ml) (SE)	Mean Number of Servings (SE)	*n* ^a^	*N* ^b^	Mean Daily SSB Consumption (ml) (SE)	Mean Number of Servings (SE)
35–44 y	248	6,537,458	529.4 (53.1)	1.49 (0.15)	400	8,744,682	385.1 (26.8)	1.08 (0.08)
45–54 y	237	6,953,571	463.2 (33.4)	1.30 (0.09)	277	6,519,086	336.2 (33.4)	0.95 (0.09)
55–64 y	184	4,053,496	418.1 (67.4)	1.18 (0.19)	225	4,593,913	203.1 (32.9)	0.57 (0.09)
65–74 y	214	2,857,241	325.1 (57.9)	0.92 (0.16)	239	2,671,939	219.2 (24.6)	0.62 (0.07)
75–94 y^c^	137	1,869,730	245.7 (48.3)	0.69 (0.14)	177	2,006,184	186.0 (22.8)	0.52 (0.06)
Total	1,020	22,271,496	438.4 (24.5)	1.24 (0.07)	1,318	24,535,804	303.7 (15.0)	0.86 (0.04)

A serving is a portion of 355 ml. SSBs included industrialized flavored waters, sodas, *aguas frescas*, *horchatas*, chocolate water, fruit and vegetable juices, and sports and energy drinks.

^a^ *n* represents the number of individuals included in the survey sample.

^b^ *N* represents the number of people in the Mexican population in 2012 within the stated age and gender stratum.

^c^Age deciles 75–84 y and 85–94 y are collapsed due to the small numbers of individuals aged 85–94 y who were surveyed by ENSANUT 2012 (*n* = 32 for men aged 85–94 y; *n* = 24 for women aged 85–94 y).

ENSANUT, Encuesta Nacional de Salud y Nutrición; SE, standard error; SSB, sugar-sweetened beverage.

A population-wide 10% reduction in SSB intake (with 39% calorie compensation) is projected to reduce the number of new cases of diabetes by 189,300 (95% UI 155,400–218,100) for the time period 2013 to 2022, a 4.9% lower incidence than what is projected when assuming no change in SSB consumption (Table 2). The resulting reduction in diabetes burden would amount to a total savings in direct healthcare costs incurred by diabetes patients of about 983 million international dollars (95% UI $769 million–$1.2 billion) over 10 y (Table 2). Varying the degree of calorie compensation, a 10% reduction in SSBs could decrease the 10-y cumulative incidence by as few as 66,000 cases (95% UI 39,800–91,600), if 100% of calories reduced through lower SSB consumption were replaced by calories from other sources, or as many as 265,100 cases (95% UI 222,100–304,100), if all calories reduced through lower SSB consumption were translated into weight change. A 20% reduction in SSB intake with 39% calorie compensation would result in approximately 9.5% fewer incident diabetes cases than projected when assuming no change in SSB consumption, for a total savings of nearly 1.9 billion international dollars (95% UI $1.5 billion–$2.3 billion).

**Table 2:** Number of incident cases of diabetes prevented and diabetes healthcare costs saved under intervention scenarios compared to the base case scenario among Mexican adults aged 35–94 y for 2013–2022.

Intervention Scenario	Number of Incident Diabetes Cases Prevented			Diabetes Healthcare Cost Savings		
	*n*	95% UI	Percent Change from Base Case^a^	Cost Savings (Millions of International Dollars)^b^	95% UI	Percent Change from Base Case^c^
**10% decrease in SSB consumption**						
100% calorie replacement	66,000	39,800–91,600	−1.7%	$483	$290–$673	−0.4%
39% calorie replacement	189,300	155,400–218,100	−4.9%	$983	$769–$1,173	−0.8%
0% calorie replacement	265,100	222,100–304,100	−6.8%	$1,289	$1,040–$1,510	−1.1%
**20% decrease in SSB consumption**						
100% calorie replacement	130,900	84,000–180,700	−3.4%	$956	$616–$1,326	−0.8%
39% calorie replacement	368,600	309,700–425,500	−9.5%	$1,916	$1,529–$2,304	−1.6%
0% calorie replacement	514,800	438,700–586,000	−13.2%	$2,503	$2,075–$2,923	−2.2%

The base case simulation results serve as the comparator for all intervention scenarios and assume no change in SSB consumption from consumption levels measured in ENSANUT 2012. Six intervention scenarios are presented, with two levels of SSB consumption lowering (10% and 20% decline) and three levels of calorie compensation: 100%, 39%, and 0% of calories replaced.

^a^Percent decline is relative to the number of incident cases of diabetes projected by a model simulation that assumes no change in SSB consumption; the base case simulation projects a cumulative incidence of diabetes totaling *n* = 3,888,500 for 2013–2022.

^b^Amount of savings is for direct healthcare costs for diabetes patients expressed in millions of 2012 international dollars.

^c^Percent decline is relative to direct healthcare costs associated with diabetes projected under the assumption of no change in SSB consumption; the base case simulation projects a total cost for diabetes for 2013–2022 of 116,213 million international dollars.

ENSANUT, Encuesta Nacional de Salud y Nutrición; SSB, sugar-sweetened beverage; UI, uncertainty interval.

**Fig 1:**
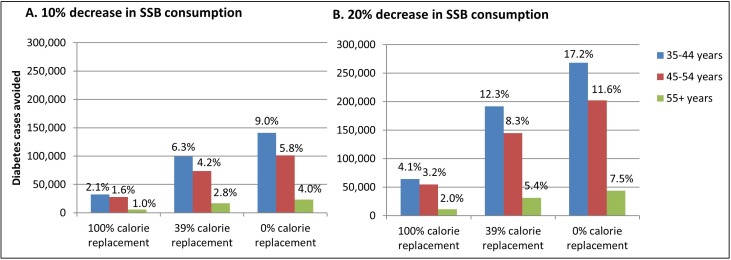
Incident cases of diabetes prevented during 2013–2022 under different intervention scenarios, with results for each scenario stratified by age group. Results for intervention simulations assuming a 10% decrease (A) or 20% decrease (B) in SSB intake and varying levels of calorie compensation. The *y*-axis values represent the absolute number of diabetes cases prevented by the intervention compared to the base case simulation, which assumes no change in SSB consumption. The base case simulation projects a 10-y cumulative incidence of diabetes of 1,565,600 for individuals aged 35–44 y, 1,741,600 for individuals aged 45–54 y, and 580,900 for individuals aged 55–94 y. The labels on each bar represent the percent of the total number of incident cases predicted by the base case simulation that is reduced under the intervention assumptions. SSB, sugar-sweetened beverage.

The effect of decreasing SSB intake on diabetes incidence and associated costs would be greater for men than women regardless of scenario (S2 Table). The youngest age group modeled (ages 35–44 y) had the largest absolute and relative reductions in diabetes incidence, with roughly half of all cases averted over the 10-y simulation occurring in this age group (Fig 1; S3 Table).

**Fig 2:**
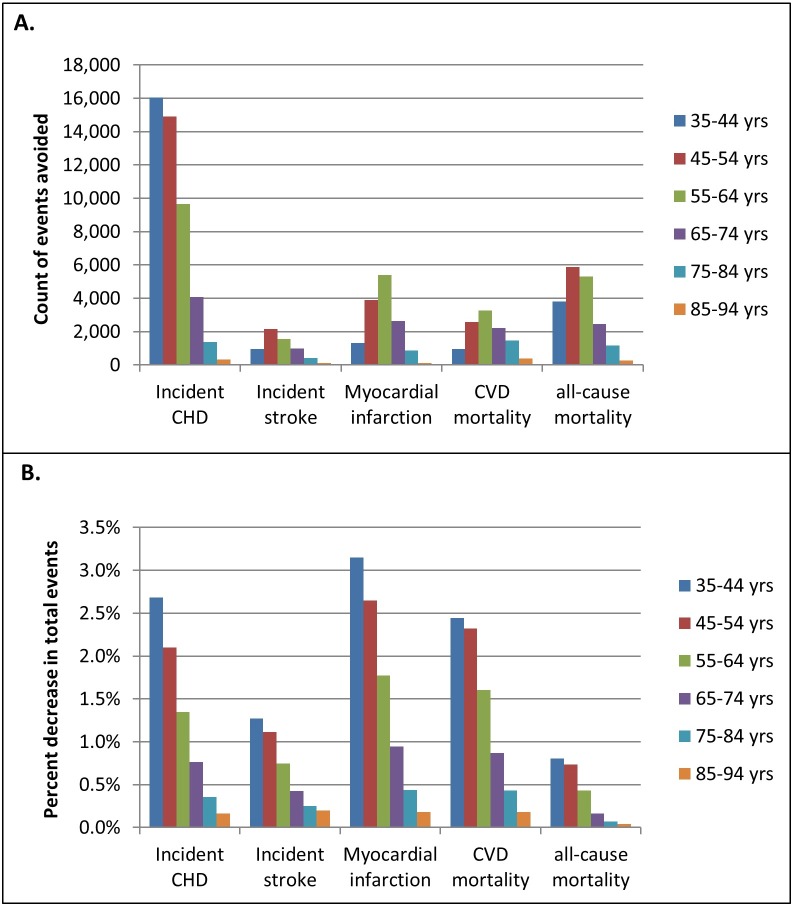
Age-stratified decrease in cardiovascular disease events and deaths predicted for a 10% reduction in sugar-sweetened beverage consumption with 39% caloric compensation, compared to a base case simulation assuming no change in consumption. (A) Total events and deaths prevented over 10 y under the intervention scenario. (B) The fraction of the total events predicted under the base case scenario that are prevented under the intervention scenario. The total counts of events and deaths for each age decile under the base case scenario and the change in events under the 10% and 20% SSB reduction scenarios are presented in S4 Table. CHD, coronary heart disease; CVD, cardiovascular disease.

In addition to reducing diabetes, declines in SSB consumption would lower the number of CVD events and deaths (Table 3). A 10% reduction in SSB consumption with 39% calorie compensation would lower incident CHD cases by approximately 46,300 (95% UI 40,900–51,800), stroke by 6,200 (95% UI 4,600–8,100), CHD and stroke deaths by 10,900 (95% UI 9,400–12,500), and deaths from all causes by 18,900 (95% UI 15,500–22,700). The projected decreases in CVD events and deaths over the 10-y simulation were distributed unequally over age groups (Fig 2). For all CVD and mortality outcomes, the events avoided with reduced SSB consumption were concentrated among those under 65 y of age (Fig 2A); the percent decrease in events relative to the base case (which assumes no change in SSB consumption) was highest among individuals aged 35–44 y and decreased progressively with each increasing age group (Fig 2B; S4 Table).

**Table 3:** Total number of cardiovascular disease events and deaths avoided over 10 y among Mexican adults aged 35–94 y under different assumptions about sugar-sweetened beverage consumption reduction and replacement with calories from other sources.

Outcome	Base Case Total Number	10% Reduction in SSB Consumption			20% Reduction in SSB Consumption		
		100% Calorie Replacement	39% Calorie Replacement	0% Calorie Replacement	100% Calorie Replacement	39% Calorie Replacement	0% Calorie Replacement
Incident CHD^a^	3,144,000	5,100 (1,900–8,100)	46,300 (40,900–51,800)	72,100 (64,300–79,800)	10,000 (4,500–15,800)	90,900 (80,500–101,100)	141,500 (126,700–155,400)
Incident stroke	936,400	1,800 (600–2,900)	6,200 (4,600–8,100)	9,000 (6,800–11,500)	3,500 (1,600–5,900)	12,300 (9,300–16,300)	17,800 (13,700–23,100)
Myocardial infarction^b^	1,041,300	1,500 (500–2,300)	14,200 (12,600–15,900)	22,200 (19,800–24,600)	2,900 (1,300–4,700)	27,900 (24,700–31,000)	43,400 (38,600–47,500)
CHD mortality	929,700	900 (300–1,400)	9,300 (8,200–10,400)	14,500 (13,000–16,000)	1,700 (700–2,900)	18,200 (16,100–20,200)	28,400 (25,200–31,100)
Stroke mortality	237,700	400 (200–700)	1,600 (1,200–2,100)	2,300 (1,700–3,000)	900 (400–1,500)	3,200 (2,400–4,200)	4,600 (3,500–6,000)
All-cause mortality	6,419,000	4,200 (1,900–6,400)	18,900 (15,500–22,700)	28,100 (23,700–33,100)	8,300 (4,200–12,700)	37,100 (30,700–43,900)	55,200 (46,300–64,200)

Data given as number (95% uncertainty interval).

^a^Includes angina, myocardial infarction, arrest, ischemic heart disease, and heart failure.

^b^Includes new and recurrent myocardial infarctions.

CHD, coronary heart disease; SSB, sugar-sweetened beverage.

Cardiovascular disease and mortality results were sensitive to assumptions about the level of SSB reduction and degree of calorie compensation (Tables 3 and S5). For example, depending on the range of calorie compensation (from 100% [all calories replaced] to 0% [no calories replaced]), a 10% decrease in SSB consumption may prevent as few as 1,500 myocardial infarctions (95% UI 500–2,300) or as many as 22,200 myocardial infarctions (95% UI 19,800–24,600) (Table 3). Assuming a higher CVD event rate and mortality rate associated with diabetes for Mexicans compared with rates predicted from Framingham Heart Study data resulted in somewhat higher estimates for events averted (S6 Table).

## Discussion

Mexico’s SSB consumption has increased in recent years such that SSB calories now account for a substantial proportion of overall caloric intake [2]. Our population modeling of CVD and diabetes in Mexico suggests that if Mexico’s SSB tax leads to population-wide reductions in SSB intake, as suggested by declines in SSB purchases already observed since the initiation of the tax [11], the policy will have a profound impact on disease burden in Mexico. We project that substantial health gains and cost savings are likely to result from reduced SSB consumption stemming from the current 10% excise tax, particularly among younger Mexicans, who are the highest consumers of these beverages.

The recent acceleration of obesity and diabetes rates among Mexicans—particularly young Mexicans—has led to a rapid increase in the chronic disease burden associated with diabetes and its complications. Our main policy simulations estimate that the SSB tax alone could prevent 189,300 cases of diabetes and save about 983 million international dollars in direct healthcare costs attributable to diabetes over the time period 2013–2022, with the overwhelming majority of the benefit occurring among adults under the age of 55 y. Because of the high consumption of SSBs and prevalence of obesity, as well as the young population structure, Mexico can expect a 2.6-fold larger diabetes rate reduction compared to simulations of SSB taxation in the United States using nearly identical assumptions over the same timeframe [39,40]. In addition to a reduction in diabetes incidence, we also observed reductions in CVD events and deaths with SSB tax simulations, with the reduced burden again occurring most prominently among the younger age groups. Preventing new cases of diabetes and CVD among young Mexicans will yield substantial additional benefits over a life course, as measured by years of life lived free of these chronic conditions; these health benefits are not fully captured by our 10-y simulations and highlight that our estimates are likely a conservative description of the true health benefits of this intervention. Comparisons of similar policy strategies in different population contexts highlight the importance of understanding the demographics and epidemiology of a particular population when choosing a public health strategy for maximal impact. This comparison also underscores the large anticipated health benefits from policies aimed at obesity and diabetes in Mexico, where the obesity and diabetes epidemic is particularly striking.

Our estimates are likely conservative. We modeled the impact of reduced SSB consumption for those 35 y of age and older due to limitations in epidemiologic data for CVD for those of younger ages. Given the high consumption of SSBs and prevalence of obesity in children and young adults [1,2], a larger impact might be expected as the younger generation ages. While our estimate of non-CVD deaths captured deaths from diabetes-related complications like renal disease, we did not capture changes in event rates for diabetic nephropathy and end-stage renal disease. These complications may be particularly common in Mexico and represent additional health burdens that may improve with reduced SSB consumption.

For calculations of healthcare costs, we included direct costs of hospital days and outpatient consultations for those with diabetes and estimated the savings that can be expected over the 10 y following intervention. Due to data limitations, we did not take into consideration the incremental savings from preventing CHD, stroke events, chronic kidney disease, or other conditions not captured in hospital records under diabetes codes. This likely resulted in substantial underestimation of savings, given that CVD and chronic kidney disease currently rank among the most prominent contributors to financial burden and causes of mortality in Mexico [46,47]. We also did not measure the savings that would result from the reduced need for healthcare infrastructure to deliver prevention and management services for diabetes and associated complications. Others have reported on the inadequacy of the current Mexican healthcare system to address the rising rate of diabetes, citing low rates of screening and limited capacity for chronic disease management [48]. In addition to the direct economic benefits we project in our modeling, the SSB tax might be expected to yield indirect cost savings associated with having a healthier workforce and reduced out-of-pocket expenditures absorbed by individual households [49,50]. Prior research suggests that indirect social and productivity costs could represent between 52% and 55% of the total cost of diabetes and CVD [8,49,51]. Finally, we included only revenue associated with reductions in healthcare utilization, while the revenue generated by the tax itself was not considered. Tax revenue that is invested in additional efforts to reduce diabetes risk would be expected to additionally increase the health benefits modeled here. Indeed, the Mexican government has planned to allocate a portion of SSB tax revenue to programs aimed at preventing and controlling diabetes as well as programs aimed at increasing access to drinkable water in schools and public spaces (including installation and maintenance of drinking water fountains) and decreasing undernutrition [52,53], which could magnify the health benefits described here.

The 10% SSB tax is the cornerstone of Mexico’s National Strategy for the Prevention and Control of Overweight, Obesity, and Diabetes. Though our findings suggest that the tax could bring considerable health and economic benefits, large and sustained declines in SSB consumption will likely require a combination of strategies including mass media campaigns, healthy food consumption subsides, nutritional labeling, and marketing restrictions, in addition to taxation [54]. The Mexican government has implemented other initiatives that could enhance the effects of the tax, including school-based guidelines for serving healthy foods and beverages, mandatory front-of-package labeling, and regulations around marketing foods and beverages to children [43]. We chose to focus on the SSB tax because it is a national policy of global interest and has a sufficient research base to define its effects on consumption and health. We conducted additional simulations reflecting declines in consumption larger than those expected from the current tax in order to understand what might be achieved from additional public health interventions.

The strengths of our projections include the use of a validated CVD simulation model adapted to describe the population of Mexico, using multiple Mexican national data sources to define inputs on SSB consumption and post-tax change in SSB consumption and the occurrence of diabetes and CVD outcomes. However, the study has several limitations. We defined SSBs according to methods developed by the Global Burden of Disease Study 2010 [55]. It is possible to include or exclude other beverages in this group, and misclassification could have led us to over- or underestimate average pre-tax SSB consumption in Mexican adults [1,2]. SSB consumption is based primarily on self-report, a measure that has been shown by others to likely underestimate consumption, again supporting the likely conservative nature of our estimates of potential health benefits. For our main simulations, we assumed that consumers reducing their consumption of SSBs would replace 39% of SSB calories with calories from other beverage types, but this assumption was based on reports from the US and may not apply equally in the Mexican population. Nonetheless, we tested the sensitivity of our finding and still found an important health benefit when varying the percentage of caloric compensation from other beverages and, importantly, found significant health benefits even under the assumption that all of the calories from SSB consumption are replaced. Only one elasticity parameter linking taxation with downstream effects on consumption was considered, an assumption that was validated by Mexican market research surveys [11]. However, as the same study showed, price elasticity varies by socioeconomic status and geographic location, with larger reductions in purchasing among poorer households [11]. The average effect we report here is likely to be heterogeneous across population subgroups, with greater effects in cities and among poor individuals. Importantly, while lower-income populations may bear a disproportionate burden from taxation, they are also likely to accrue the health and economic benefits from prevention of diabetes and CVD [11]. Finally, we model theoretical reductions in consumption based on taxation; one industry-funded study—limited by the generalizability of the study sample and interpretation of results—reported minimal impact of the SSB tax in Mexico [56], but a larger study reporting on a representative population in Mexico found substantial reductions in purchases following the initiation of the tax that are compatible with the effects we model here [11].

In conclusion, our findings suggest that Mexico’s SSB tax has the potential to substantially cut back SSB consumption, reducing downstream diabetes and CVD morbidity and mortality. Healthcare savings resulting from reduced SSB consumption could be reallocated toward other public health promotion programs and toward the augmentation of personnel and infrastructure to improve care for diabetes, CVD, and other diseases.

## Supporting Information

S1 Appendix Supporting information for the Cardiovascular Disease Policy Model–Mexico structure, inputs and calibration, and simulation assumptions. (DOCX)

S1 Table Daily volume in milliliters of sugar-sweetened beverages consumed per person per day among Mexican adults estimated from the 2012 Mexican National Health and Nutrition Survey (ENSANUT). (DOCX)

S2 Table Cumulative number of avoided events and deaths (percent difference) in three different scenarios of reduced sugar-sweetened beverage consumption (10%, 20%, and 40% reduction) for 2013–2022 among Mexican adults aged 35–94 y, reported separately for men and women. (DOCX)

S3 Table Cumulative number of incident cases of diabetes avoided and diabetes cost savings for 2013–2022 under two scenarios of sugar-sweetened beverage consumption reduction (10% and 20%; both with 39% calorie compensation), stratified by age decile. (DOCX)

S4 Table Cumulative number of cardiovascular disease events and deaths avoided for 2013–2022 under two scenarios of sugar-sweetened beverage consumption reduction (10% and 20%; both with 39% calorie compensation), stratified by age decile. (DOCX)

S5 Table Cumulative number of incident diabetes cases, cardiovascular disease events, and deaths prevented for 2013–2022 among Mexican adults aged 35–94 y, assuming a 40% reduction in sugar-sweetened beverage consumption and 39% caloric compensation. (DOCX)

S6 Table Cumulative number of cardiovascular disease events and deaths avoided for 2013–2022 among Mexican adults aged 35–94 y under two sets of assumptions about the association between diabetes and the risk of coronary heart disease, stroke, and non-cardiovascular disease death. (DOCX)

S1 File Cardiovascular Disease Policy Model software commons developer project participation agreement. (DOC)

## Abbreviations

BMI: body mass index
CHD: coronary heart disease
CVD: cardiovascular disease
CVDPM: Cardiovascular Disease Policy Model
ENSANUT: Encuesta Nacional de Salud y Nutrición
SBP: systolic blood pressure
SE: standard error
SINAIS: Sistema Nacional de Información en Salud
SSB: sugar-sweetened beverage
UI: uncertainty interval
