# Supplementary material for: Projected Impact of Mexico’s Sugar-Sweetened Beverage Tax Policy on Diabetes and Cardiovascular Disease: A Modeling Study
Source: PLoS Med. 2016 Nov 1;13(11):e1002158. doi: 10.1371/journal.pmed.1002158 (PMC5089730; doi:10.1371/journal.pmed.1002158)
Supplement: S1 Appendix — (DOCX) [file pmed.1002158.s001.docx]

S1 Appendix. Supporting information for the Cardiovascular Disease Policy Model – Mexico structure, inputs and calibration and simulation assumptions

Contents

Detailed structure of the CVD Policy Model-Mexico 2

Data Sources 4

Risk functions for incident diabetes, CHD, stroke and Non-CVD death 6

Transition between risk factors 8

Population Impact Fraction 8

Model Calibration………………………………………………………………………..9

Estimation of direct healthcare costs associated with type 2 diabetes 9

Simulation input assumptions 11

Appendix tables and figures 13

References 21

### **Detailed structure of the CVD Policy Model-Mexico**

The Cardiovascular Disease (CVD) Policy Model (CVDPM) is a computer simulation, state transition (Markov cohort) model that estimates future diabetes incidence and CVD (coronary heart disease and stroke) prevalence, incidence, mortality and costs in the population age 35 to 94 years old (**Appendix Figure A)**. The CVDPM-Mexico is a version of the CVD Policy Model that uses Mexico-specific inputs wherever possible and is calibrated to within <1% of the total number of CVD events and deaths in 2010 as reported in national data (**Appendix Figure B**) [1].

The model is composed of 3 submodels: the demographic-epidemiological submodel, the bridge submodel, and the disease history submodel (**Appendix Figure A**)). The *demographic-epidemiological submodel* predicts type 2 diabetes, coronary heart disease (CHD) and stroke incidence and non-CVD mortality in the population without CVD stratified by age (six 10 year age groups spanning from 35 to 94 years old), sex, and six modifiable risk factors categorized by up to 3 levels. The six risk factors included in the CVDPM-Mexico are:

- Body mass index (BMI): <25 kg/m^2^, 25 kg/m^2^ to 29.9 kg/m^2^ or >30 kg/m^2^
- Systolic blood pressure (SBP): <120 mmHg, 120 mmHg to 139.9 mmHg, or ≥140 mmHg
- Low density lipoprotein cholesterol level (LDLc): <100 mg/dl, 100 mg/dl to 129.9 mg/dl, or ≥130mg/dl
- High density lipoprotein cholesterol level (HDLc): <40mg/dl, 40mg/dl to 59.9mg/dl, or ≥60mg/dl
- Smoking status: active smoker, non-smoker with exposure to environmental tobacco smoke, or non-smoker without environmental exposure
- Type 2 Diabetes Mellitus (T2DM): with or without T2DM

Each year, based on the multivariate distribution of risk factors, the model estimates the fraction of the diabetes-free population that develops type 2 diabetes as well as the fraction of the CVD-free population that develops coronary heart disease or stroke. The subgroup with incident CVD moves into *the bridge submodel*, where the initial event type and its sequelae over the first 30 days are identified. For incident coronary heart disease, events are designated as angina, myocardial infarction (MI), or cardiac arrest. The *disease history submodel* estimates subsequent MI and stroke events, coronary revascularization procedures, and CVD and non-CVD mortality in the population with a history of CVD, stratified by prior CVD state, age group and sex. The CVD states include: angina only, MI only, stroke only, and Stroke + MI. Each year, there is a probability of having a repeat event and/or transitioning to a new CVD state. When inputs are provided, the model has the capability of computing annual costs and quality of life-year adjustments associated with each CVD state.

The model can be used to forecast CVD trends and simulate interventions through the modification of input data including population structure and dynamics, risk factor distributions, coefficients defining the relationship between risk factors and disease incidence, event and case fatality rates, costs, and quality –of life adjustments.

### CVDPM-Mexico Data Sources

#### A detailed list of inputs for the CVDPM-Mexico can be found in Appendix Table A, described further below.

#### Population

Data for the baseline population was from the most recent national population census, conducted in 2010 by the National Institute of Statistics and Geography in Mexico (INEGI) [2]. Data on estimated population from 2010 to 2050 were obtained from the population projections calculated in 2013 and reported by the “*Consejo Nacional de Población*” (CONAPO) [3].

#### Risk factor means and frequencies

Data on Mexican population risk factor distributions came from two sources. Estimates for BMI, SBP, LDLc, HDLc and T2DM were obtained from the 2006 *Encuesta Nacional de Salud y Nutrición* (ENSANUT) (Mexican National Health and Nutrition Survey) [4]. ENSANUT is a nationally representative, cross-sectional, multi-stage, cluster sampling survey designed to obtain health and nutrition information from the Mexican population. Prevalence of tobacco usage was obtained from the 2009 *Encuesta global de tabaquismo en adultos* (GATS)), a nationally representative survey of the Mexican population ≥15 years of age designed to gather data on smoking behavior in the adult population [5].

#### CVD incidence

Incidence of CHD and stroke was estimated from the 2010 hospitalization databases of the *Instituto Mexicano del Seguro Social* (IMSS) (the Mexican Social Security Institute)[6], the Instituto de Seguridad y Servicios Sociales de los Trabajadores del Estado (ISSSTE) (the State Employees’ Social Security and Services Institute) [7], and the Ministry of Health (SS) via the *Sistema Nacional de Información en Salud* (SINAIS) webpage hospitalization discharge information [1,8]. All the data were classified according to the International Classification of Diseases, 10^th^ revision (ICD10) codes [9] to categorize CHD hospitalizations (I20-I25, I46 and I50-I51) and stroke hospitalizations (I60-I69), and incidence was further stratified by age decile and sex.

#### CVD Mortality

Data regarding CVD deaths were obtained from the SINAIS 2010 general mortality data [8]. This source provides the most accurate report of vital statistics for the Mexican population. Mortality data were stratified by age decile, sex, ICD10 code, and place of death.

CHD deaths were calculated as the sum of (I20-I25, I46) +2/3*(I149+I50+I51). Total stroke deaths were estimated by adding up the deaths from the ICD10 codes I60 to I69.

**Chronic CHD** We classified CHD deaths as resulting from chronic CHD if it was reported on the death certificate that the death occurred on the streets, at home, or in another location other than the hospital or if the place of death was not known*.*

**Total of Non-CVD deaths.** Non-CVD deaths were estimated as the difference between the sum of CHD and stroke deaths, and total deaths reported in vital statistics (SINAIS) [8].

National in-hospital events and deaths

We obtained detailed hospitalization information, including in-hospital events and deaths, from the three major health services providers – SS, IMSS, and ISSSTE [6,7,10]. For other health service institutions including Mexican Petroleum (PEMEX, Petroleos Mexicanos), Army (Defensa Nacional), Navy (Marina), and other public and private hospital systems, we obtained total CHD deaths and total stroke deaths from SINAIS [8]. To obtain events and case fatality estimates for the smaller health organizations, we applied the ratio of CHD and stroke deaths observed in the three large health organizations to the smaller organizations.

#### Cardiac arrest (sudden death)

The total number of cardiac arrests in the population was computed from the difference between the total number of CHD deaths (ICD-10 codes I20-I25) recorded in the national mortality statistics that occurred in hospital setting and the total of in-hospital CHD deaths (ICD-10 code: I20-I25) from the hospitalization data bases. We then added to this value to the total number of hospitalizations coded for cardiac arrest (ICD-10 code I46) from the in-hospital discharge data.

Arrest survival to hospital: We calculated the probability of surviving an arrest to hospitalization as the total number hospitalizations coded with ICD-10 code I46 divided by total cardiac arrests.

### Risk functions for incident diabetes, CHD, stroke and Non-CVD death

The incidence of outcomes (i.e., type 2 diabetes, CHD, stroke, and non-CVD death) in each risk factor cell for the at-risk population were determined by a risk function incorporating age/sex specific alphas and risk factor specific betas$\left\{\beta_{k}\right.,k=1,2,3,...,6\}$, which are constant over the time span of a simulation, and cell specific risk factor means $\left\{m_{k},k=1,2,3,...,6\}\right.$, which are altered by user-defined intervention. The risk function is defined as:

$$r=e^{(\alpha+\sum_{k=1}^{6} \beta_{k}m_{k})}/(1+e^{(\alpha+\sum_{k=1}^{6} \beta_{k}m_{k})})$$

β-coefficients for incident CHD, stroke and non-CVD deaths were estimated using data from the Framingham Heart Study Original Cohort (exam 13-28) and Offspring Cohort (exam 1-7) [11-14] and the counting process extension of the Cox proportional hazards model, which allows for time-dependent covariates [15].The diabetes incidence function is also estimated using Framingham data, with adjustments for the association between BMI and age based on published data demonstrating a lower effect of BMI on diabetes in older ages [16]. Multivariate models for incident CHD included as covariates SBP, LDL, smoking, HDL, and diabetes; models for stroke and for non-CVD death included SBP, smoking, and diabetes. Models for incident type 2 diabetes include only BMI. Coefficients generated from Framingham data have been reported to be useful in different populations [17-19]. We estimated overall incidence of diabetes, CHD, stroke and non-CVD death by age range and sex for 2010 by adjusting the Framingham incidence estimates to account for the differences in risk factor distribution in the Mexico population as measured by ENSANUT 2006. We estimated the corresponding values of the intercepts by iterative fitting of the risk function to the overall incidence. We assumed all risks and rates to be constant over time. The risk functions are applied to every state in every year of a simulation to accommodate the competing risk for all outcomes naturally over time.

We assumed that all the risk factors modify the incidence of angina, infarction and arrest equally, except for smoking. Regarding excess risk from tobacco, we assumed that smokers had a higher risk for arrest and myocardial infarction and a lower excess risk for angina [20]. Environmental tobacco exposure had a 1.26 relative risk compared to non-exposed non-smokers just for cardiac arrest and myocardial infarction [21].

###

### Transition between risk factors

We included annual probabilities of transition from one risk factor stratum to another to maintain age- and gender-specific risk factor proportions of the population describe in ENSANUT 2006 [4]. The annual transition probabilities were calculated by an iterative algorithm, taking into account the competing risk effect of the Model’s CHD incidence and non-CVD death rates established in the base case.

### Population Impact Fraction

We estimated the number of new cases of diabetes that would be prevented if current levels of SSB consumption are lowered by a percentage expected from the implementation of a national soda tax [22]. Percentage reductions in SSB intake were translated into absolute changes in risk factor means for each age and gender stratum, with status quo consumption calculated using data from ENSANUT 2012 [23]. The resulting value comparing incident diabetes in the hypothetical intervention to that of the base case is known as the population impact fraction (PIF), defined as [24,25]:

$$PIF=\frac{\int_{i=1}^{max} PiRRi-\int_{i=1}^{max} Pi^{'}RRi}{\int_{i=1}^{Max} P\text{i}{RR}_{i}}$$

### where *i* corresponds to various SSB consumption values as continuous variable; RR_i_ is the fitted relative risk for developing diabetes at exposure level *i*, P_i_ is the base SSB intake distribution; and P_i_’ represents the post reduction in consumption distribution. The resulting PIF therefore represents the relative reduction in diabetes incidence as a result of the percentage reduction in SSB intake intervention scenario.

### Model Calibration

The CVD Policy Model Mexico was calibrated to national data on the number of CVD events and deaths [1]. The appropriate denominator for event and mortality rates is the population at risk, but the size of the population at risk changes in the course of a year due to events (post AMI event rates are higher) and mortality. Initial rates are calculated based on the population at risk at the beginning of the year and are adjusted iteratively using updated population-at-risk estimates from simulations with the prior rates. Iterations were terminated when all 2010 model events and deaths came within 1% of total events and deaths observed in national data for each age and gender stratum. A summary of calibration results are presented in **Appendix Figure B**.

### Estimation of direct healthcare costs associated with type 2 diabetes

We calculated direct health expenditures for type 2 diabetes in Mexico following the methodology described previously [26,27]. The calculation combined 2011 expenditure data from Mexico’s *Sistema de Cuentas de Salud en México* (SICUENTAS) (System of Health Accounts) [28], 2011service use data for each of the three primary health public institutions (IMSS, ISSSTE and Ministry of Health) [6,7,10], and population estimates of diagnosed diabetes prevalence from ENSANUT 2012 [23] to generate a mean expenditure person with diabetes in Mexico in 2011 for each age decile. Detailed steps were as follows:

1. National healthcare expenditure data from SICUENTAS [28] were used to obtain the total healthcare expenditures in 2011 for generalist outpatient consultation visits, specialist outpatient consultation visits and hospitalizations for each of the three primary healthcare institutions serving the Mexican population (IMSS, ISSSTE, and the Ministry of Health).
2. Hospitalization and outpatient utilization databases [6,7,10] were used to calculate the total volume of each type of service (counts of general outpatient visits, specialist outpatient visits, and hospitalization days) for each healthcare institution.
3. The average cost of a generalist visit, specialist visit, and day of hospitalization in 2011 was then computed for each healthcare institution by dividing the institution-specific total expenditure per service (step 1) by the total volume of service provided in 2011 (step 2).
4. The healthcare utilization databases [6,7,10] were then used to calculate the average number of generalist visits, specialty care visits and hospitalization days for patients with diabetes in 2011. Patients with diabetes were identified as those with utilization records coded with ICD-10 codes E11-E14 listed as the primary or secondary diagnosis in the utilization records. Average service use for diabetics was stratified by age decile and by healthcare institution (IMSS, ISSSTE, and the Ministry of Health).
5. For each healthcare institution and age strata, the average cost of each type of visit or hospitalization day (step 3) was multiplied by the average number of visits or days for each diabetic in 2011 (step 4). These values were then summed over service use type for each institution to generate an average cost of healthcare service use in 2011 for each diabetic, stratified by age decile.
6. Expenditures were then scaled to the national level using data from ENSANUT 2012 [23] on age-specific diabetes prevalence and healthcare affiliation. For each decile, we estimated the total number of diabetics in Mexico receiving care at IMSS, ISSSTE, and the Ministry of Health.
7. The institution-specific average healthcare expenditures per diabetic in 2011 (step 5) were multiplied by the total number of diabetics affiliated with the given institution (step 6). For each age stratum, these total health expenditures for diabetics were then summed over the three institutions to generate a total expenditure for type 2 diabetic patients in 2011 in Mexican pesos (overall total of $54 billion Mexican pesos). (**Appendix Table B**).
8. Total expenditures in Mexican pesos (step 7) were then converted into per-person annual expenditures by dividing by the total population in each age strata receiving services at IMSS, ISSSTE, or the Ministry of Health as measured by ENSANUT 2012 [23], thus generating a weighted average using the proportion of diabetics receiving care at the given healthcare institution as the weight. (Appendix Table S3 “Expenditure per capita in 2011 (MXN pesos))
9. Mean expenditure per diabetic in 2011 (step 8) was then converted into 2011 international dollars using a purchasing power parity rate of 7.67 Mexican pesos = $1.00 U.S. international dollar, and then inflated to 2012 international dollars using an inflation rate of $3.57% [29].

### Simulation input assumptions

Expanding on information in the main manuscript, **Appendix Table C** presents a detailed list of variables and assumptions used for simulations forecasting the impact of SSB taxation in Mexico. The assumed relationship between changes in SSB consumption and changes in cardiovascular disease risk factors and outcomes are further depicted in **Appendix Figure C**.

### Appendix tables and figures

| S1 Appendix Table A. Data sources used for CVD Policy Model- Mexico inputs | |
| --- | --- |
| **Variable** | **Data Sources** |
| Mexican population 2010 | Instituto Nacional de Estadística y Geografía (INEGI) [2] [National Institute of Statistics and Geography] |
| Population projection estimates 2010-2050 | Consejo Nacional de Población (CONAPO) [3]  [National Population Council] |
| Total and cause-specific mortality 2010 | Sistema Nacional de Información en Salud (SINAIS) [1]  [National Health Information System] |
| CHD incidence 2010 | Instituto Mexicano del Seguro Social (IMSS) [1,6]  [Mexican Social Security Institute]  Instituto de Seguridad y Servicios Sociales de los Trabajadores del Estado (ISSSTE) [1,7][The State’s Employees Social Security and Services Institute]  Sistema Nacional de Información en Salud (SINAIS) [8] [Ministry of Health, National Health Information System] |
| CHD Events 2010 | Instituto Mexicano del Seguro Social (IMSS) [1,6]  [Mexican Social Security Institute]  Instituto de Seguridad y Servicios Sociales de los Trabajadores del Estado (ISSSTE) [1,7] [The State’s Employees Social Security and Services Institute]  Sistema Nacional de Información en Salud (SINAIS) [8] [Ministry of Health, National Health Information System] |
| Risk of CVD events and non-CVD deaths | Framingham Heart Study Original Cohort and Offspring Cohort [11-14] |
| Risk factors distribution | Encuesta Nacional de Salud y Nutrición 2006 (ENSANUT) [4] [Health and Nutrition Survey 2006]  Encuesta Global de Tabaquismo en Adultos (GATS) [5] [Smoking Adult Survey 2009] |
| Disease Prevalence | Encuesta Nacional de Salud y Nutrición 2006 (ENSANUT) [4] [Health and Nutrition Survey 2006] |

| **S1 Appendix Table B.**  Calculation of direct healthcare expenditures per person with type 2 diabetes annually in the Mexican population of adults 35 to 94 years of age. | | | | |
| --- | --- | --- | --- | --- |
| **Age groups (years)** | **Total expenditure in 2011 for type 2 diabetics* (MXN pesos)** | **Expenditure per capita in 2011 (MXN pesos)** | **Expenditure per capita in 2011 (international dollars)^**^** | **Expenditure per capita in 2012 (international dollars)** † |
|  |  |  |  |  |
| 35-44 | $5,517,867,332.41 | $7,796.69 | $1016.2 | $1054.3 |
| 45-54 | $12,704,670,651.40 | $8,413.26 | $1096.6 | $1135.7 |
| 55-64 | $16,329,048,715.18 | $9,666.19 | $1259.9 | $1304.8 |
| 65-74 | $11,600,114,076.05 | $9,772.05 | $1273.7 | $1319.1 |
| 75-84 | $6,130,272,013.57 | $12,917.38 | $1683.6 | $1743.7 |
| 85-94 | $1,626,789,663.33 | $13,031.46 | $1698.5 | $1759.1 |
| **Total 35-94** | **$53,908,762,451.95** |  |  |  |
| * Healthcare expenditures include direct healthcare costs associated with outpatient generalist visits, outpatient specialty care visits, and hospitalizations for patients with type 2 diabetes (defined with ICD-10 codes E11-E14) receiving services in one of the three primary healthcare institutions serving the Mexican population (IMSS, ISSSTE, and the Ministry of Health)  ** Exchange rate used 1 US International dollar = 7.67 MXN pesos  † Cost inflated costs from 2011 a 3.57% to adjust costs to 2012 US | | | | |

| **S1 Appendix Table C.** Summary of variables used for the forecasting of the effect of SSB taxation in Mexico on diabetes, cardiovascular diseases, and mortality outcomes | | | |
| --- | --- | --- | --- |
| **Variable** | **Input estimate** | **95% confidence interval for inputs varied in Monte Carlo simulations** | **Data source** |
| Consumer Price Elasticity | -1.16 (0.02) |  | Colchero et al. [22] |
| Reduction in consumption by a 10% tax per 1lt in SSB | 10.6%-11.6% |  | Colchero et al. [22] |
| Baseline average intake of SSB  (ml/day) | 438.4 ml/day (35-94 males)  303.7 ml/day(35 to 94 female) |  | Estimated from the data collected by 24hr dietary recall from ENSANUT 2012 [23] |
| Beverage caloric compensation by a SSB reduction in consumption | 39% | (Note: uncertainty evaluated with separate scenario analyses assuming 0% and 100% calorie compensation) | Assuming this percentage is composed by water (1/3), milk or juices (1/3), and diet beverages (1/3) [30] |
| Relative risk (RR) of excess SSB consumption on incident diabetes | RR=1.19 | (1.09, 1.31) | Imamura et al. (2015) [31]; published values adjusted for definition of serving size |
| Beta for the association between a 1-unit increase in BMI and incident diabetes | Decreases over age;  Beta=0.161 for 55-64 year olds | (-0.035, 0.357)  for 55-64 year olds | Framingham Heart Study data [11-14]  Biggs et al. (2010) [16] |
| Changes in systolic blood pressure due to a reduction in SSB consumption of 1 serving/day | *Men: -*0.78 mmHg  *Women:* -0.61 mmHg | *Men*: (-1.47, -0.9)  *Women*: (-1.48, 0.27)) | Chen L et al. [32] |
| Changes in systolic blood pressure per 1 unit increase in BMI* | *Men*: 1.43 mmHg  *Women:* 1.24 mm Hg |  | Wilsgaard et al.[33] |
| Changes in LDL per 1 unit increase in BMI | *Men:* 2.75 mg/dL  *Women*: 2.24 mg/dL |  | Wilsgaard et al.[34] |
| Changes in HDL per 1 unit increase in BMI | *Men:* 1.55 mg/dL  *Women*: 0.77 mg/dL |  | Wilsgaard et al.[34] |
| Increased risk of CHD associated with diabetes** | RR=1.84 | (1.59, 2.14) | Framingham Heart Study data [11-14] |
| Beta for the association of SBP and incident CHD** | Decreases over age; beta=0.013 for 60 year old | (0.010, 0.016)  for 60 year old | Framingham Heart Study data [11-14] |
| Increased risk of stroke associated with diabetes*** | RR=2.31 | (1.82, 2.93) | Framingham Heart Study data [11-14] |
| Beta for the association of SBP with incident stroke*** | Decreases over age; beta=0.019 for 60 year old | (0.013, 0.025)  for 60 year old | Framingham Heart Study data [11-14] |
| Increased risk of non-CVD mortality associated with diabetes**** | Decreases over age;  RR=1.57 for 60 year old | (1.32, 1.87)  for 60 year old | Framingham Heart Study data [11-14] |
| Beta for the association of SBP with non-CVD mortality**** | Decreases over age;  Beta=0.00468 for 60 year old | (0.00172, 0.00764)  for 60 year old | Framingham Heart Study data [11-14] |
| Mean annual direct health expenditure of diabetes | $604-1011 USD varied from 35 to 94 years respectively |  | Data for the Instituto Mexicano del Seguro Social (IMSS), Instituto de Seguridad y Servicios Sociales de los Trabajadores del Estado (ISSSTE), and Ministry of Health collected from theSistema Nacional de Información en Salud (SINAIS) [28] |
| Discount rate | 3% |  | Assumption |
| **Abbreviations:** CHD: coronary heart disease; CVD: cardiovascular disease; SBP: systolic blood pressure; LDL: Low-density lipoprotein; HDL: high-density lipoprotein  ** Models included the following variables: diabetes, SBP, LDL,HDL, and smoking; assumed age interaction for SBP and diabetes  *** Models included the following variables: SBP, smoking and diabetes; assumed age interactions with SBP  **** Models included the following variables: SBP, smoking and diabetes; assumed age interactions with SBP and diabetes | | | |


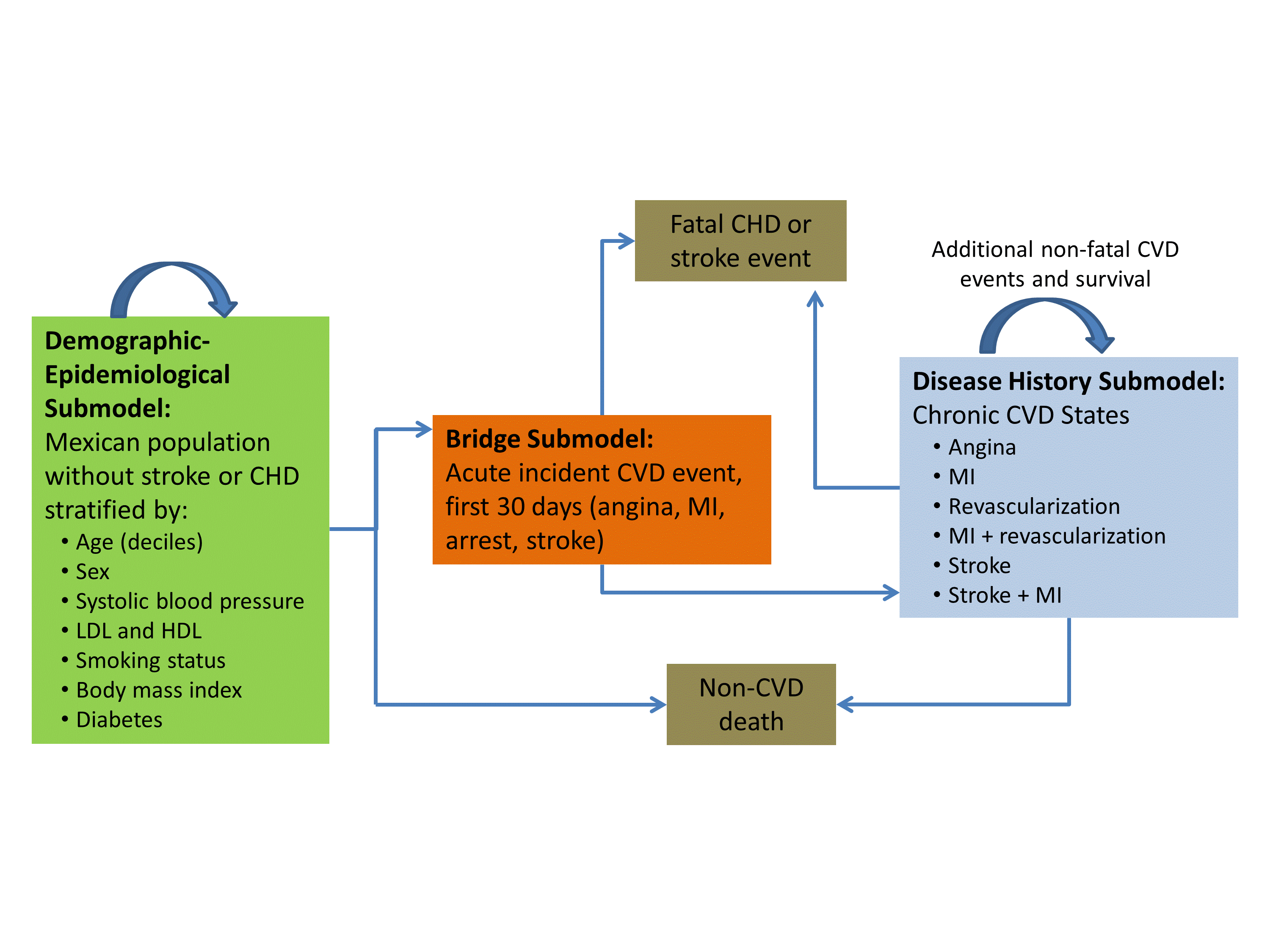


S1 Appendix Figure A. Cardiovascular Disease Policy Model – Mexico structure and disease states. The Cardiovascular Disease Policy Model divides the population into those that have no history of cardiovascular disease (the Demographic-Epidemiological submodel), those with a new CHD event (the Bridge Submodel), and those with a history of cardiovascular disease (Disease History Submodel). Each annual cycle, subgroups defined by levels age, sex, and cardiovascular risk factors in the Demographic-Epidemiological Submodel have a probability of transitioning between risk factor groups, having a first CVD event and moving into the Bridge Submodel, or of non-CVD death. Those with a history of cardiovascular disease have an annual probability of transitioning between disease states or to a mortality state. The current analysis involved 10 annual cycles of the model, thus providing estimates of 10-year cumulative incidence of outcomes including type 2 diabetes, CHD, stroke, myocardial infarctions, and mortality.

**S1 Appendix Figure B. Comparison between the reported national incidence of CVD events and CHD deaths calculated with the CVD Policy Model Mexico for 2010.**


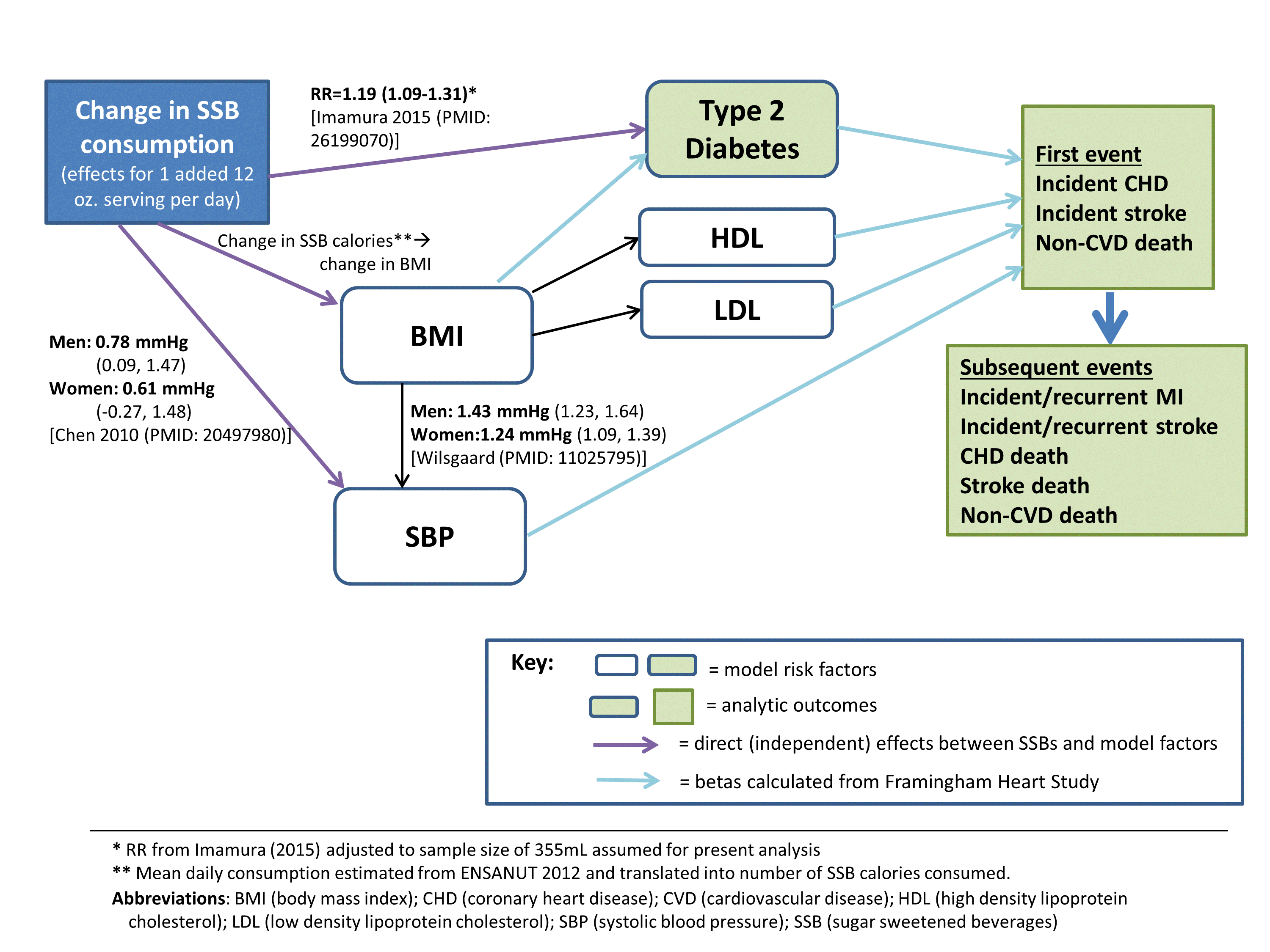


**S1 Appendix Figure C.** Directed acyclic graph describing the relationship between changes in SSB consumption and risk factors and outcomes in the CVD Policy Model –Mexico For model simulations, changes in SSB consumption were assumed to have direct and independent effects on model risk factors including BMI, SBP, and type 2 diabetes as well as indirect effects on SBP, diabetes, HDL and LDL that were mediated through BMI. Inputs for translating changes in SBP into changes in risk factors were based on published literature (see S1 Appendix Table C). Framingham Heart Study data were used to determine the risk functions defining the relationships between BMI and diabetes as well as all risk factors and CVD and mortality outcomes. In this analysis (and in the CVD Policy Model in general), type 2 diabetes is both a risk factor connected to downstream CVD events and deaths as well as an outcome in and of itself. A detailed depiction of the CVD Policy Model – Mexico is shown in S1 Appendix Figure A.

### References

### 1. Dirección General de Información a la Salud. Bases de datos sobre defunciones. Defunciones generales (INEGI/SALUD): 2010. Secretaría de Salud. 2012 [cited 2016 Aug 11]. Available: http://www.dgis.salud.gob.mx/contenidos/basesdedatos/std_defunciones.html.

### 2. Intituto Nacional de Estadística y Geografía. Estadística población, hogares y vivienda. Intituto Nacional de Estadística y Geografía; 2014 [cited 2014 Jan 20]. Available: http://www3.inegi.org.mx/sistemas/temas/default.aspx?s=est&c=17484.

### 3. Secretaría de Gobernación. Proyecciones de la población 2010–2050. Secretaría de Gobernación. 2014 [cited 2014 Jan 20]. Available: http://www.conapo.gob.mx/es/CONAPO/Proyecciones.

### 4. Olaiz-Fernández G, Rivera-Dommarco J, Shamah-Levy T, Rojas R, Villalpando-Hernández S, Hernández-Ávila M. Encuesta Nacional de Salud y Nutrición 2006 Cuernavaca, México: Instituto Nacional de Salud Pública; 2006. Available from: http://ensanut.insp.mx/informes/ensanut2006.pdf. Accessed: January 20, 2014.

### 5. Reynales Shigematsu L, Shamah T, Méndez I, Rojas R, Lazcano E. Encuesta Global de Tabaquismo en Adultos: México 2009. Organización Panamerica de la Salud. 2010 [cited 2014 Jan 20]. Available: http://www.who.int/tobacco/surveillance/gats_rep_mexico.pdf.

### 6. Instituto Mexicano del Seguro Social. Memorias estadística 2011. Instituto Mexicano del Seguro Social. 2012 [cited 2016 Jun 14]. Available: http://www.imss.gob.mx/conoce-al-imss/memoria-estadistica-2011.

### 7. Instituto de Seguridad Social y Servicios para los Trabajadores del Estado. Estadística: anuarios 2011. Instituto de Seguridad Social y Servicios para los Trabajadores del Estad. 2012 [cited 2016 Jun14]. Available: http://www.issste.gob.mx/datosabiertos/anuarios/anuarios2011.html.

### 8. Secretaría de Salud. Cubos Dinámicos-Defunciones (Mortalidad 2012). Secretaría de Salud. 2014 [cited 2016 Sept 27]. Available: http://www.dgis.salud.gob.mx/contenidos/basesdedatos/bdc_defunciones_gobmx.html.

### 9. World Health Organization. ICD-10 Version: 2010. World Health Organization. 2010 [cited 2016 Sept 27]. Available: http://apps.who.int/classifications/icd10/browse/2010/en.

### 10. Sistema Nacional de Información en Salud. Direccion General de Información en Salud. Bases de Sector Salud.Cubos Dinamicos-Egresos Hospitalarios 2012. Mexico: Secretaría de Salud 2012 [cited 2016 Sept 27]. Available: http://www.dgis.salud.gob.mx/contenidos/basesdedatos/bdc_egresoshosp_gobmx.html.

### 11. Framingham Heart Study-Cohort (FHS-Cohort) data request site. Biologic Specimen and Data Repository Information Coordinating Center: National Heart, Lung, and Blood Institute. Available from: http://biolincc.nhlbi.nih.gov/studies/framcohort/?q=framingham. Accessed: April 2, 2013.

### 12. Framingham Heart Study-Offspring Study (FHS-OS) data request site. Biologic Specimen and Data Repository Information Coordinating Center: National Heart, Lung, and Blood Institute. Available from: http://biolincc.nhlbi.nih.gov/studies/framoffspring/?q=framingham. Accessed: April 2, 2013.

### 13. Dawber TR. The Framingham Study: the epidemiology of atherosclerotic disease. Cambridge, MA: Harvard University Press; 1980.

### 14. Feinleib M, Kannel WB, Garrison RJ, McNamara PM, Castelli WP. The Framingham Offspring Study. Design and preliminary data. Prev Med. 1975 Dec;4(4):518-25. PMID: 1208363.

### 15. Kleinbaum DG, Klein M. Survival analysis: a self-learning text. Chapter 8. New York, NY: Springer; 2012. p. 366-79.

### 16. Biggs ML, Mukamal KJ, Luchsinger JA, Ix JH, Carnethon MR, Newman AB, et al. Association between adiposity in midlife and older age and risk of diabetes in older adults. Jama. 2010 Jun 23;303(24):2504-12. PMID: 20571017. PMCID: PMC3047456.

### 17. D'Agostino RB, Sr., Grundy S, Sullivan LM, Wilson P. Validation of the Framingham coronary heart disease prediction scores: results of a multiple ethnic groups investigation. JAMA : the journal of the American Medical Association. 2001 Jul 11;286(2):180-7. PMID: 11448281.

### 18. Liu J, Hong Y, D'Agostino RB, Sr., Wu Z, Wang W, Sun J, et al. Predictive value for the Chinese population of the Framingham CHD risk assessment tool compared with the Chinese Multi-Provincial Cohort Study. JAMA : the journal of the American Medical Association. 2004 Jun 2;291(21):2591-9. PMID: 15173150.

### 19. Brindle P, Emberson J, Lampe F, Walker M, Whincup P, Fahey T, et al. Predictive accuracy of the Framingham coronary risk score in British men: prospective cohort study. BMJ (Clinical research ed). 2003 Nov 29;327(7426):1267. PMID: 14644971. PMCID: 286248.

### 20. Parish S, Collins R, Peto R, Youngman L, Barton J, Jayne K, et al. Cigarette smoking, tar yields, and non-fatal myocardial infarction: 14,000 cases and 32,000 controls in the United Kingdom. The International Studies of Infarct Survival (ISIS) Collaborators. BMJ (Clinical research ed). 1995 Aug 19;311(7003):471-7. PMID: 7647641. PMCID: 2550542.

### 21. Law MR, Morris JK, Wald NJ. Environmental tobacco smoke exposure and ischaemic heart disease: an evaluation of the evidence. BMJ (Clinical research ed). 1997 Oct 18;315(7114):973-80. PMID: 9365294. PMCID: 2127675.

### 22. Colchero MA, Salgado JC, Unar-Munguia M, Hernandez-Avila M, Rivera-Dommarco JA. Price elasticity of the demand for sugar sweetened beverages and soft drinks in Mexico. Econ Hum Biol. 2015 Sep 5;19:129-37. PMID: 26386463.

### 23. Gutiérrez J, Rivera-Dommarco J, Shamah-Levy T, Villalpando-Hernández S, Franco A, Cuevas-Nasu L, et al. Encuesta Nacional de Salud y Nutrición 2012: resultados nacionales. Instituto Nacional de Salud Pública. 2012 [cited 2014 Jan 20]. Available: http://ensanut.insp.mx/informes/ENSANUT2012ResultadosNacionales.pdf.

### 24. Drescher K, Becher H. Estimating the generalized impact fraction from case-control data. Biometrics. 1997 Sep;53(3):1170-6. PMID: 9290235.

### 25. Eide GE, Heuch I. Attributable fractions: fundamental concepts and their visualization. Stat Methods Med Res. 2001 Jun;10(3):159-93. PMID: 11446147.

### 26. Burgos LÁ, Hurtado LC, editors. Cuentas en diabetes mellitus, enfermedades cardiovasculares y obesidad: México 2006. Secretaría de Salud. 2009 [cited 2016 Jul 10]. Available: http://www.insp.mx/images/stories/Produccion/pdf/101203_cdiabetes.pdf.

### 27. Villalobos Hernández A, Ham Chande R. Gasto por diabetes en el sistema público de salud en México, 2010-2030. Mexico: Colegio de Mexico (COLMEX). 2014 [cited 2016 July 10]. Available: <http://colmex.alma.exlibrisgroup.com/view/delivery/52COLMEX_INST/1264973160002716>.

28. Secretaría de Salud. Boletín de información estadística. Volumen IV. Recursos financieros. Numero 31, Año 2011. Secretaría de Salud. 2012 [cited 2016 Jul 10]. Available: http://www.dgis.salud.gob.mx/contenidos/publicaciones/p_bie.html.

### 29. Instituto Nacional de Estadística y Geografía. Calculadora de inflación. Instituto Nacional de Estadística y Geografía. 2014 [cited 2014 Jan 1]. Available: http://www.inegi.org.mx/sistemas/indiceprecios/calculadorainflacion.aspx.

### 30. Stookey JD, Constant F, Gardner CD, Popkin BM. Replacing sweetened caloric beverages with drinking water is associated with lower energy intake. Obesity (Silver Spring). 2007 Dec;15(12):3013-22. PMID: 18198310.

### 31. Imamura F, O'Connor L, Ye Z, Mursu J, Hayashino Y, Bhupathiraju SN, et al. Consumption of sugar sweetened beverages, artificially sweetened beverages, and fruit juice and incidence of type 2 diabetes: systematic review, meta-analysis, and estimation of population attributable fraction. BMJ (Clinical research ed). 2015;351:h3576. PMID: 26199070. PMCID: PMC4510779.

### 32. Chen L, Caballero B, Mitchell DC, Loria C, Lin PH, Champagne CM, et al. Reducing consumption of sugar-sweetened beverages is associated with reduced blood pressure: a prospective study among United States adults. Circulation. 2010 Jun 8;121(22):2398-406. PMID: 20497980. PMCID: 2892032.

### 33. Wilsgaard T, Schirmer H, Arnesen E. Impact of body weight on blood pressure with a focus on sex differences: the Tromso Study, 1986-1995. Archives of internal medicine. 2000 Oct 9;160(18):2847-53. PMID: 11025795.

### 34. Wilsgaard T, Arnesen E. Change in serum lipids and body mass index by age, sex, and smoking status: the Tromso study 1986-1995. Ann Epidemiol. 2004 Apr;14(4):265-73. PMID: 15066606.
