# Supplementary material for: Projected Impact of Mexico’s Sugar-Sweetened Beverage Tax Policy on Diabetes and Cardiovascular Disease: A Modeling Study
Source: PLoS Med. 2016 Nov 1;13(11):e1002158. doi: 10.1371/journal.pmed.1002158 (PMC5089730; doi:10.1371/journal.pmed.1002158)
Supplement: S1 Table — (DOCX) [file pmed.1002158.s002.docx]

| **S1 Table.** Daily volume in millilitres of sugar sweetened beverages consumed per person per day among Mexican adults estimated from the 2012 Mexican National Nutrition and Health Survey | | | | | | | | |
| --- | --- | --- | --- | --- | --- | --- | --- | --- |
|  | **Average daily consumption among men**  **(mL/day*)** | | | | **Average daily consumption among women**  **(mL/day*)** | | | |
| **Age group** | **Median consumption** | **Quartile 1** | **Quartile 3** | **Maximum** | **Median consumption** | **Quartile 1** | **Quartile 3** | **Maximum** |
| **35-44** | 470 | 0 | 734 | 4913 | 257 | 0 | 564 | 2646 |
| **45-54** | 451 | 0 | 648 | 3000 | 226 | 0 | 559 | 2820 |
| **55-64** | 347 | 0 | 658 | 2500 | 0 | 0 | 338 | 1901 |
| **65-74** | 240 | 0 | 451 | 2412 | 130 | 0 | 376 | 2100 |
| **75-94** | 0 | 0 | 352 | 3404 | 75 | 0 | 300 | 1180 |
| **Total population 35-94 years** | **346** | **0** | **595** | **4913** | **226** | **0** | **500** | **2820** |
| ***** Milliliters of SSBs consumed per day were translated into servings per day for model inputs assuming 1 serving = 355mL | | | | | | | | |
