# Supplementary material for: Projected Impact of Mexico’s Sugar-Sweetened Beverage Tax Policy on Diabetes and Cardiovascular Disease: A Modeling Study
Source: PLoS Med. 2016 Nov 1;13(11):e1002158. doi: 10.1371/journal.pmed.1002158 (PMC5089730; doi:10.1371/journal.pmed.1002158)
Supplement: S2 Table — (DOCX) [file pmed.1002158.s003.docx]

| **S2 Table.** Cumulative number of avoided events and deaths (% difference*) in three different SSB reductions in consumption scenarios (10%, 20% and 40%)** from 2013-2022 among Mexican adults 35-94 years old, reported separately for men and women. | | | | |
| --- | --- | --- | --- | --- |
|  |  | **Events prevented (% change from base case*****)** | | |
|  | **Base case** events** | **10% reduction in SSB consumption***** | **20% reduction in SSB consumption***** | **40% reduction in SSB consumption***** |
| **Men** | | | | |
| Incident Type 2 Diabetes | 1,945,000 | 108,500 (-5.6%) | 210,300 (-10.8%) | 402,600 (-20.7%) |
| Incident CHD† | 1,837,300 | 33,400 (-1.8%) | 65,300 (-3.6%) | 128,200 (-7.0%) |
| Incident Stroke | 471,400 | 4,000 (-0.9%) | 7,900 (-1.7%) | 15,500 (-3.3%) |
| Myocardial Infarctions^‡^ | 649,400 | 11,500 (-1.8%) | 22,400 (-3.5%) | 43,900 (-6.8%) |
| CHD mortality | 522,500 | 7,200 (-1.4%) | 14,000 (-2.7%) | 27,400 (-5.2%) |
| Stroke mortality | 120,100 | 1,000 (-0.9%) | 2,000 (-1.7%) | 4,000 (-3.4%) |
| All-cause mortality | 3,427,100 | 13,800 (-0.4%) | 26,900 (-0.8%) | 52,800 (-1.5%) |
| **Women** | | | | |
| Incident Type 2 Diabetes | 1,943,000 | 80,900 (-4.2%) | 158,300 (-8.1%) | 280,100 (-14.4%) |
| Incident CHD† | 1,306,700 | 12,900(-1.0%) | 25,700 (-2.0%) | 49,600 (-3.8%) |
| Incident Stroke | 465,000 | 2,200 (-0.5%) | 4,400 (-0.9%) | 8,400 (-1.8%) |
| Myocardial Infarctions^‡^ | 391,800 | 2,700 (-0.7%) | 5,400 (-1.4%) | 10,600 (-2.7%) |
| CHD mortality | 407,200 | 2,100(-0.5%) | 4,200 (-1.0%) | 8,100 (-2.0%) |
| Stroke mortality | 117,600 | 500 (-0.5%) | 1,100 (-0.9%) | 2,100 (-1.7%) |
| All-cause mortality | 2,991,900 | 5,100 (-0.2%) | 10,300 (-0.3%) | 19,500 (-0.7%) |
| * % change in the number of events under the intervention scenario as compared to base case simulations that assume no change in SSB consumption  ** All base case results (counts and total costs) are from simulations that assume no change in SSB consumption  *** all scenarios assume a 39% calorie compensation level  † CHD: coronary heart disease, it includes angina, myocardial infarction, arrest, ischaemic heart disease, heart failure  ^‡^  Total myocardial infarctions includes new and recurrent myocardial infarctions | | | | |
