# Supplementary material for: Projected Impact of Mexico’s Sugar-Sweetened Beverage Tax Policy on Diabetes and Cardiovascular Disease: A Modeling Study
Source: PLoS Med. 2016 Nov 1;13(11):e1002158. doi: 10.1371/journal.pmed.1002158 (PMC5089730; doi:10.1371/journal.pmed.1002158)
Supplement: S3 Table — (DOCX) [file pmed.1002158.s004.docx]

| **S3 Table**. Cumulative number of incident cases of diabetes avoided and diabetes cost savings from 2013-2022 under two assumptions of SSB reduction (10% and 20%; both with 39% calorie compensation), stratified by age decile | | | | | | |
| --- | --- | --- | --- | --- | --- | --- |
|  |  |  | **10% SSB reduction** | | **20% SSB reduction** | |
| **Incident cases of diabetes** | **Age** | **Base case**† **count** | **Events prevented** | **% change*** | **Events prevented** | **% change*** |
|  | **35-44** | 1,565,600 | 99,000 | -6.3% | 192,100 | -12.3% |
|  | **45-54** | 1,741,600 | 73,800 | -4.2% | 145,100 | -8.3% |
|  | **55-64** | 575,500 | 16,400 | -2.9% | 31,100 | -5.4% |
|  | **65-74** | 3,100 | 100 | -3.3% | 200 | -6.0% |
|  | **75-84** | 1,600 | 0 | -1.5% | 100 | -3.4% |
|  | **85-94** | 600 | 0 | -1.4% | 0 | -3.3% |
| **Diabetes costs**  **(millions of international dollars)** |  | **Base case**† **total costs**** | **Cost savings**** | **% change*** | **Cost savings**** | **% change*** |
|  | **35-44** | $19,479 | $292 | -1.5% | $566 | -2.9% |
|  | **45-54** | $30,823 | $455 | -1.5% | $891 | -2.9% |
|  | **55-64** | $34,748 | $216 | -0.6% | $420 | -1.2% |
|  | **65-74** | $20,209 | $25 | -0.1% | $48 | -0.2% |
|  | **75-84** | $9,247 | $4 | 0.04% | $8 | 0.1% |
|  | **85-94** | $1,708 | $1 | 0.03% | $1 | 0.1% |
| † All base case results (counts and total costs) are from simulations that assume no change in SSB consumption  * % change in the number of events under the intervention scenario as compared to base case simulations that assume no change in SSB consumption  ** Base case total costs and cost savings comparing intervention scenario costs to base case totals are expressed in millions of international dollars. | | | | | | |
