# Supplementary material for: Projected Impact of Mexico’s Sugar-Sweetened Beverage Tax Policy on Diabetes and Cardiovascular Disease: A Modeling Study
Source: PLoS Med. 2016 Nov 1;13(11):e1002158. doi: 10.1371/journal.pmed.1002158 (PMC5089730; doi:10.1371/journal.pmed.1002158)
Supplement: S4 Table — (DOCX) [file pmed.1002158.s005.docx]

| **S4 Table**. Cumulative number of cardiovascular disease events and deaths avoided from 2013-2022 under two assumptions of SSB reduction (10% and 20%) with 39% calorie compensation, stratified by age decile | | | | | | |
| --- | --- | --- | --- | --- | --- | --- |
|  |  |  | **10% SSB reduction** | | **20% SSB reduction** | |
| **Outcome** | **Age** | **Base case* events** | **Events prevented** | **% change**** | **Events prevented** | **% change**** |
| **Incident CHD**† | **35-44** | 598,400 | 16,000 | -2.7% | 31,500 | -5.3% |
|  | **45-54** | 709,300 | 14,900 | -2.1% | 29,200 | -4.1% |
|  | **55-64** | 716,700 | 9,700 | -1.4% | 18,900 | -2.6% |
|  | **65-74** | 533,300 | 4,100 | -0.8% | 8,100 | -1.5% |
|  | **75-84** | 382,000 | 1,400 | -0.4% | 2,700 | -0.7% |
|  | **85-94** | 204,400 | 300 | -0.2% | 700 | -0.3% |
| **Incident stroke** | **35-44** | 76,200 | 1,000 | -1.3% | 1,900 | -2.5% |
|  | **45-54** | 192,700 | 2,200 | -1.1% | 4,200 | -2.2% |
|  | **55-64** | 210,400 | 1,600 | -0.8% | 3,100 | -1.5% |
|  | **65-74** | 228,500 | 1,000 | -0.4% | 1,900 | -0.9% |
|  | **75-84** | 168,600 | 400 | -0.3% | 900 | -0.5% |
|  | **85-94** | 60,100 | 100 | -0.2% | 200 | -0.4% |
| **Myocardial infarction**^‡^ | **35-44** | 41,800 | 1,300 | -3.215% | 2,600 | -6.2% |
|  | **45-54** | 146,900 | 3,900 | -2.7% | 7,500 | -5.1% |
|  | **55-64** | 303,600 | 5,400 | -1.8% | 10,500 | -3.5% |
|  | **65-74** | 277,800 | 2,600 | -0.9% | 5,200 | -1.9% |
|  | **75-84** | 202,600 | 900 | -0.4% | 1,800 | -0.9% |
|  | **85-94** | 68,400 | 100 | -0.2% | 200 | -0.4% |
| **CHD mortality** | **35-44** | 20,400 | 700 | -3.4% | 1,400 | -6.6% |
|  | **45-54** | 70,900 | 2,100 | -2.9% | 4,000 | -5.7% |
|  | **55-64** | 153,600 | 2,900 | -1.9% | 5,600 | -3.6% |
|  | **65-74** | 197,900 | 2,000 | -1.0% | 3,900 | -2.0% |
|  | **75-84** | 293,700 | 1,300 | -0.5% | 2,700 | -0.9% |
|  | **85-94** | 193,200 | 300 | -0.2% | 700 | -0.4% |
| **Stroke mortality** | **35-44** | 18,300 | 300 | -1.4% | 500 | -2.7% |
|  | **45-54** | 40,000 | 500 | -1.2% | 1,000 | -2.4% |
|  | **55-64** | 49,800 | 400 | -0.8% | 800 | -1.6% |
|  | **65-74** | 58,400 | 300 | -0.5% | 500 | -0.9% |
|  | **75-84** | 52,900 | 200 | -0.4% | 400 | -0.7% |
|  | **85-94** | 35,600 | 100 | -0.3% | 200 | -0.6% |
| **All-cause mortality** | **35-44** | 473,200 | 3,800 | -0.8% | 7,400 | -1.6% |
|  | **45-54** | 803,600 | 5,900 | -0.7% | 11,500 | -1.4% |
|  | **55-64** | 1,227,000 | 5,300 | -0.4% | 10,400 | -0.9% |
|  | **65-74** | 1,493,300 | 2,500 | -0.2% | 4,900 | -0.3% |
|  | **75-84** | 1,713,400 | 1,200 | -0.1% | 2,300 | -0.1% |
|  | **85-94** | 708,500 | 300 | -0.04% | 500 | -0.1% |
| * All base case results (counts and total costs) are from simulations that assume no change in SSB consumption  ** % change in the number of events under the intervention scenario as compared to base case simulations that assume no change in SSB consumption  † CHD: coronary heart disease, it includes angina, myocardial infarction, arrest, ischaemic heart disease, heart failure  ^‡^  Total myocardial infarctions includes new and recurrent myocardial infarctions | | | | | | |
