# Supplementary material for: Projected Impact of Mexico’s Sugar-Sweetened Beverage Tax Policy on Diabetes and Cardiovascular Disease: A Modeling Study
Source: PLoS Med. 2016 Nov 1;13(11):e1002158. doi: 10.1371/journal.pmed.1002158 (PMC5089730; doi:10.1371/journal.pmed.1002158)
Supplement: S5 Table — (DOCX) [file pmed.1002158.s006.docx]

| **S5 Table**. Cumulative number of diabetes, cardiovascular disease events, and deaths prevented from 2013-2022 among Mexican adults 35-94 years of age, assuming a 40% reduction in SSB consumption and 39% caloric compensation | | | |
| --- | --- | --- | --- |
|  | **base case* events** | **count averted** | **% change**** |
| **Incident Type 2 Diabetes** | 3,888,500 | 682,300 | -17.5% |
| **Incident CHD†** | 3,144,000 | 177,800 | -5.7% |
| **Incident Stroke** | 936,400 | 23,900 | -2.6% |
| **Myocardial Infarctions^‡^** | 1,041,300 | 54,400 | -5.2% |
| **CHD mortality** | 929,700 | 35,500 | -3.8% |
| **Stroke mortality** | 237,700 | 6,100 | -2.6% |
| **All-cause mortality** | 6,419,000 | 72,300 | -1.1% |
| * All base case results (counts and total costs) are from simulations that assume no change in SSB consumption  ** % change in the number of events under the intervention scenario as compared to base case simulations that assume no change in SSB consumption  † CHD: coronary heart disease, it includes angina, myocardial infarction, arrest, ischaemic heart disease, heart failure  ^‡^  Total myocardial infarctions includes new and recurrent myocardial infarctions | | | |
