# Supplementary material for: Projected Impact of Mexico’s Sugar-Sweetened Beverage Tax Policy on Diabetes and Cardiovascular Disease: A Modeling Study
Source: PLoS Med. 2016 Nov 1;13(11):e1002158. doi: 10.1371/journal.pmed.1002158 (PMC5089730; doi:10.1371/journal.pmed.1002158)
Supplement: S6 Table — (DOCX) [file pmed.1002158.s007.docx]

| **S6 Table.** Cumulative number of cardiovascular disease events and deaths avoided from 2013-2022 among Mexican adults age 35-94 with a 10% reduction in SSB consumption** under two sets of assumptions about the association between diabetes and the risk of coronary heart disease, stroke and non-cardiovascular disease death. | | | | |
| --- | --- | --- | --- | --- |
|  | **Main analysis assumption***  (Betas from Framingham data) | | **Alternative scenario***  (Assumes higher beta on diabetes in Mexican adults) | |
| **Outcome** | **Baseline events** | **Events avoided****  **(% change from baseline)** | **Baseline events** | **Events avoided****  **(% change from baseline)** |
| **Incident CHD†** | 3,144,000 | 46,300 (-1.5%) | 3,144,400 | 51,600 (-1.6%) |
| **Incident Stroke** | 936,400 | 6,200 (-0.7%) | 933,000 | 7,000 (-0.8%) |
| **Myocardial Infarctions^‡^** | 1,041,300 | 14,200 (-1.4%) | 1,035,000 | 16,200 (-1.6%) |
| **CHD mortality** | 929,700 | 9,300 (-1.0%) | 929,000 | 10,700 (-1.2%) |
| **Stroke mortality** | 237,700 | 1,600 (-0.7%) | 254,200 | 1,900 (-0.8%) |
| **All-cause mortality** | 6,419,000 | 18,900 (-0.3%) | 6,414,200 | 24,600 (-0.4%) |
| * For the alternative scenario, we assumed that the diabetes beta for incident coronary heart disease and non-cardiovascular disease mortality was two-fold higher among Mexican adults (RR=3.39 for incident CHD; RR varies by age for non-CVD death, RR=2.46 for 55-64 year olds) compared to the risk among members of the Framingham Offspring Study data (RR=1.84 for incident CHD; RR varies by age for non-CVD death and was RR=1.57 for 55-64 year olds), which was the source of estimates for our main simulations. For stroke, we assumed that Mexicans with diabetes compared to non-diabetics had a 3-fold increased risk (RR=3.0) of stroke, whereas our main simulations assumed RR=2.3 of stroke for diabetics compared to non-diabetics as computed from Framingham Offspring Study data.  ** Simulations assume a 10% reduction in consumption of SSBs and a 39% compensation of calories  † CHD: coronary heart disease, it includes angina, myocardial infarction, arrest, ischaemic heart disease, heart failure  ^‡^  Total myocardial infarctions includes new and recurrent myocardial infarctions | | | | |
